# Prehospital Facial Recognition Technology: Randomized Controlled Simulation Study

**DOI:** 10.5811/westjem.53140

**Published:** 2026-10-29

**Authors:** Asit Misra, Richard Cabeceiras, Abishek Sehgal, Jose L. Montero, Richard Cambo, Richard D. Rodriguez, Angel A. Brotons, Samantha L. Syms, Tulay Koru-Sengul, Eduard Tiozzo, Ivette Motola

**Affiliations:** * University of Miami Miller School of Medicine, Gordon Center for Simulation & Innovation in Medical Education, Miami, Florida; † University of Miami Miller School of Medicine, Department of Public Health Sciences, Miami, Florida; ‡ University of Miami Miller School of Medicine, Department of Physical Medicine and Rehabilitation, Miami, Florida; § University of Miami Miller School of Medicine, Department of Surgery, Miami, Florida

## Abstract

**Introduction:**

The risk of medical errors increases for the approximately 689,000 unidentified, noncommunicative patients who present to emergency departments annually. The concomitant rise in hospital admissions, lengths of stay, and mortality will likely persist unless approaches to identifying noncommunicative patients are developed and adopted. Facial recognition technology could provide a solution where scanning the face reliably provides access to identification and medical history of patients who self-enroll in the application database. Our objective was to compare paramedics’ ability to identify noncommunicative patients and make related clinical decisions using a cloud-based facial-recognition application vs standard practice.

**Methods:**

We conducted a double-blind, randomized controlled study with 90 participating firefighterparamedics in a simulated patient encounter. Our primary outcome was patient identification. Secondary outcomes included identification of key medical information, appropriateness of treatment decisions, and transport to the patient’s preferred hospital. We uploaded facial photographs and simulated medical profiles to a secure cloud-based app. Participants used a tablet to scan the patient’s face (standardized patient or actor). Successful matches returned identifying information, medical history, emergency contacts, preferred hospital, and primary care physician contact information. The 48 experimental participants had access to this patient information, while the 42 controls did not, even if they scanned the face. Standardized patients (SP) portrayed a noncommunicative urosepsis patient. Participants reported their findings to the facilitators and evaluated the technology on a 5-point Likert scale.

**Results:**

We analyzed 90 study participants (48 experimental, 42 control), of whom 40/48 (83.3%) experimental group identified the SP compared with 9/42 (21.4%) control group (OR [odds ratio], 13.87; 95% CI, 5.03–38.25; *P* < .001). Identification of important medical information of the SP by the study participants occurred in 30/48 (62.5%) in the experimental group versus 4/42 (9.5%) in control (OR, 15.83; 95% CI, 4.84–51.76; *P* < .001). Correct transport decision to the preferred hospital occurred in 27/48 (56.3%) experimental participants compared with 10/42 (23.8%) controls (OR, 4.11; 95% CI, 1.65–10.23; *P* = .02). Overall, 80% of participants reported that the technology would benefit their day-to-day clinical work.

**Conclusion:**

The use of a cloud-based facial-recognition application significantly improved firefighter-paramedics’ ability to identify noncommunicative patients and access critical medical information, with an appropriate transport decision to the patient’s preferred hospital.

## INTRODUCTION

Medical errors occur in approximately 4% to 10% of hospital patients yearly.^1^,^2^ A 2017 study reported that medical errors may have accounted for 251,000 annual deaths in the United States, making them the third leading cause of death.^3^ Although errors reported by emergency medical services (EMS) in the United Kingdom can reach as high as 13%, recent comparable data are unavailable for the US.^4^ The frequency of errors in patient care can increase in emergencies and/or when a patient’s medical history is unknown. These factors increase the likelihood of medical complications, such as misdiagnosis and over- or under-treatment.^5^

The potential for medical errors can compound when dealing with noncommunicative patients who lack identifying information.^6^ Of all medical errors in the US annually, 10% to 17% were attributed to errors in identification (ID).^7^,^8^ Additional data indicate that unknown patients comprise 0.14% to 0.54% of emergency department (ED) visits in North America.^9^,^10^ In 2018, this would have impacted between 178,000 to 689,000 patients in the US. Studies have reported that unidentified patients were mostly male (64.9% to 71%), while most patients were brought in by EMS (65.1% to 80.7%).^5^,^9^–^11^ Patients with unknown identities experienced an increased number of hospital admissions, and greater lengths of stay and incidence of mortality.^10^ Improper treatment and/or diagnostic testing resulting from a lack of patient identification can intensify when multiple unknown patients present to the ED or in mass casualty incidents, despite using electronic health records.^12^–^14^

Problems due to a lack of patient identification can also occur in routine care. In 2018, over 3.6 million individuals in. the US sought emergency treatment for a urinary tract infection (UTI), making UTI the seventh most common reason to visit the ED. A UTI is a “common cause of altered mental status, particularly in elderly patients and patients with psychiatric conditions.”^15^ This altered mental status could render patients noncommunicative and unidentifiable to healthcare professionals, causing potential delays in diagnosis and treatment.^10^,^15^ These delays in diagnosis and treatment increase morbidity and mortality because a prolonged UTI can lead to urosepsis, septic shock, or both.^16^–^18^ The challenges of unidentified patients manifest somewhat differently in prehospital versus hospital settings. In the prehospital setting, EMS clinicians must rapidly determine the likely causes of a patient’s altered mental status and choose an appropriate destination without any knowledge of the patient’s medical history. Gaining access to a patient’s records in the field could significantly help with these decisions: For example, recognizing that the patient “had frequent UTIs in the last year with the same presentation” (as opposed to having a history of multiple falls while on anticoagulation, which might be suggestive of suspected intracranial hemorrhage) would enable paramedics to make more informed differential diagnoses and destination choices, ensuring the patient is transported to the right facility. Correct identification could allow paramedics to bring a patient to the hospital or healthcare system where they usually receive care (enhancing continuity of care).

**Population Health Research Capsule**

What do we already know about this issue?

*Unidentified, noncommunicative patients have a higher risk of medical errors; facial recognition technology can serve as a reliable identification (ID) tool.*

What was the research question?

*Does a facial recognition app improve patient ID and decision-making of paramedics compared with traditional means of care for noncommunicative patients?*

What was the major finding of the study?

*Patient ID improved: 83.3% vs 21.4%; OR, 13.87 (95% CI, 5.03–38.25); P < .001. Medical information ID: 62.5% vs 9.5%; OR, 15.83 (95% CI, 4.84–51.76); P < .001.*

How does this improve population health?

*This app may improve prehospital care by facilitating quick patient ID and key medical information and supporting more appropriate transport and treatment decisions.*

In the hospital setting, an unidentified patient poses additional challenges—clinicians have no information on the patient’s allergic history, medication use, prior colonization with antibiotic-resistant organisms, or past medical history—which complicates diagnostic workup and can lead to mistreatment or delays (for example, administering a drug to which the patient is allergic or failing to consider infections in light of prior culture results). As the UTI example illustrates, not knowing a patient’s identity can precipitate medical errors or delays in care for many other conditions. Altered mental status is a common presentation of life-threatening problems such as stroke, drug overdose, or severe electrolyte imbalance, and managing these conditions is considerably more challenging when the patient is unidentified and cannot provide any history.

Access to patient information and medical history, even when a patient cannot communicate, can improve outcomes in trauma and many life-threatening diseases.^3^,^18^–^21^ A universal mechanism is urgently needed to access medical records from any location and better track patient movements throughout their medical journey.^3^,^20^–^22^ Facial recognition technology has been used in clinical settings, demonstrating a high degree of sensitivity (96.5%) and specificity (96.7%).^3^,^20^–^24^ The technology we used in this study was based on the Rekognition platform (Amazon Web Service).^25^ Currently, there is no other standard of care, and the use of facial recognition in the US healthcare system is still novel. Cloud-based facial recognition technology could be a solution that helps clinicians identify noncommunicative patients, view their medical history, and track their movements in prehospital and hospital settings.^24^

Previous studies have shown the use of simulation to validate and test a new technology or skills training.^26^–^28^ This pilot study aimed to examine the effect of a cloud-based facial recognition app on patient identification and gathering medical history among noncommunicative patients. The study also examined the effect of the same app when used by paramedics on triage, treatment, and transport decision outcomes among noncommunicative patients in a simulated environment using standardized patients (SP).

## METHODS

### Design and Population

We conducted this double-blind, randomized controlled study from May to June 2022 at the Gordon Center for Simulation and Innovation in Medical Education at the University of Miami Leonard M. Miller School of Medicine in Miami, Florida (Instiiutional Review Board approval No. 20220232).

Providing a setting that safely simulated a real-world scenario was important to understanding the implications of using facial recognition to identify patients in a prehospital setting. We used a convenience sampling method to recruit 101 local paramedics in Miami. The participants were part of a single fire-rescue–based EMS agency with approximately 800 full-time employees serving in an urban region with an approximate population of 435,000. A total of 99 paramedics provided written consent to participate and were randomly divided into a control (*n* = 50) or experimental (*n* = 49) group. Trained SPs portrayed elderly patients experiencing symptoms of urosepsis in the simulation scenario. Standardized patients were required to provide a photograph of their face. Each SP assigned to the experimental group had their patient information (for the simulated case) and photograph uploaded into the app database, ERinfo (ERinfo, Inc). Control group SPs were not in the database.

The study team included research associates, evaluators, and facilitators. Research associates consented participants, assigned study ID numbers, and conducted app training. Blinded to group assignments, evaluators gave the scenario briefing and recorded encounter durations, treatment decisions, and transport destinations. Facilitators were situated in the scenario room with the participant and the SP, available to answer questions about vital signs and physical exam findings not portrayed by the SP. Upon appropriate request, the facilitator provided emergency contact feedback.

Our primary outcome measure was patient ID. Secondary outcomes included identification of key medical information, appropriateness of treatment decisions, and transport to the preferred hospital of the patient.

### Simulation Case Scenario

The simulation case scenario was “an unidentified elderly patient with altered mental status.” The SP exhibited symptoms associated with recurrent UTI, Alzheimer disease, fever, and chills. Although responsive to verbal commands, the patient was shivering, complaining about being cold, and unable to answer identifying information. Vital signs were as follows: heart rate, 128 beats per minute; temperature, 102 °F; and respiratory rate, 24 breaths per minute. Questions about blood pressure, oxygen saturation, and blood glucose were answered with appropriate values within the reference range. A 12-lead electrocardiogram (ECG), if performed, revealed a normal sinus rhythm. Medical history included allergies to penicillin and peanuts, Alzheimer disease, hypertension, and recent admission to the University of Miami Hospital (UMH) for a UTI. Standardized patients could follow all commands but answered “I don’t know” to any identifying questions. Only experimental group participants could gather identifying information, medical history, emergency contact information, and the patient’s preferred hospital. This required the participant to choose to use the application, and subsequently, to use it correctly.

The SP encounters took place in a simulated home environment. Emergency medical serivces could not readily find identifying information because the person was alone and noncommunicative, which is a common challenge for EMS clinicians. The location was equidistant to Jackson Memorial Hospital (JMH), UMH, and Miami Veterans Affairs Medical Center. Because of the patient’s recent admission to UMH, that hospital would be ideal for further treatment. However, departmental protocol dictated that the patient be brought to JMH when no preference is provided. The paramedic could also contact the SP’s emergency contact if they successfully accessed the app, who would provide the same information provided by the app.

### Implementation and Randomization

We randomly assigned participants to the control or experimental group. Two participants at a time entered a classroom and received a tablet device containing the app. Every participant received a three-minute tutorial on app usage. Following the tutorial, the study team separated the participants, and each was directed to the appropriate briefing room for the setting and nature of the 9-1-1 call. Next, each participant went to their respective patient encounter room to assess and manage the SP. Evaluators recorded entry and exit times. The study team did not require participants to use the app. If a participant asked a facilitator whether they should use the app, the facilitator responded with, “If you want.” To ensure blinding, the study design permitted optional app use in both groups. All participants were trained on the app and had a tablet; however, the application was accessed by both groups, with successful patient identification occurring exclusively in the experimental group and no matches observed in the control group. After the scenario, participants reported their treatment interventions and transport decisions. The same SP scenario was used for all participants to ensure standardization.

A total of 99 SP encounters were conducted (50 control, 49 intervention), with a one-to-one correspondence between participant and SP encounter. Outcomes were measured based on paramedic performance during the encounter, and analyses were conducted at the participant level because each encounter reflected a single participant’s performance under identical scenario conditions. The study participants answered a Qualtrics questionnaire (Qualtrics International Inc, June 2022; Supplement 1) consisting of six demographic questions, 11 Likert-type (1 = strongly disagree, 5 = strongly agree), and one ranking question (1 = most important, 8 = least important). All study participants received a $25 gift card for participation.

Participants, evaluators, facilitators, and SPs were blinded to their study arm to address any potential source of bias. Evaluators, facilitators, and SPs were trained in their roles individually. At the beginning of each study day and at additional points during the day, the study team conducted trial runs of the scenario to ensure accuracy and script compliance.

### Data Analysis

We analyzed all data using SAS OnDemand for Academics, version 3.8 (SAS Institute Inc). Frequency and percentage distributions were calculated for all demographic outcomes (age, sex, race and ethnicity, highest level of education, and years of paramedic experience) (Table 1). Demographic outcomes of the control and experimental groups were compared using a chi-square test. We calculated odds ratios with 95% CIs to examine the differences between the two groups on binary outcomes (yes/no; calculated using the top box and the bottom box method, where “strongly disagree,” “disagree,” and “neutral” were categorized as “no,” while “agree” and “strongly agree” were categorized as “yes”). The binary outcomes included patient identification, patient contraindication identification, treatment (the participant treated the SP without causing harm while following their departmental protocol for the treatment of septic shock that included administering intravenous fluids, checking blood glucose, and administering vasopressors after two fluid boluses to achieve a mean arterial pressure of 65 mm Hg); sepsis mitigation actions (the participant provided treatments appropriate for the SP’s primary condition); treatment confidence; transport (the patient should be transported to UMH for this case); and transport confidence (participants’ belief in correct transport destination).

Mean rank with a 95% CI was used to calculate the rank of information importance to participants (1 = most important, 8 = least important) for identifying information, prior medical conditions, allergies, medications, emergency contact information, preferred language, choice of treatment facility, and primary care clinician contact information.

We calculated proportions across the groups based on the participants’ actual vs perceived confidence (what they believed) in making appropriate treatment and transport decisions. Proportions across the groups were also calculated based on the participants’ app use (ease of use, improved ability to identify patients, treat patients, identify patient contradictions, improved transport decisions, and whether the app was beneficial when evaluating patients).

We excluded 11 participants from the analyses for failure to follow the study protocol or due to opting out: Two declined to participate during the consent stage; seven were excluded due to incomplete data (partially filled out the postencounter questionnaire and left the question blank on what treatment or care provided to the patient); and two from the control group opted out immediately after their participation (SP simulation encounter) (Figure 1).

## RESULTS

We analyzed data from 90 firefighter-paramedics, 42 in the control group and 48 in the experimental group. No statistically significant demographic differences between study arms were present (Table 1).

Table 2 summarizes the achievement of desired outcomes, along with the level of confidence reported by participants with respect to having achieved outcomes. Of 48 experimental participants, 40 (83.3%) identified the patient compared with 9 of 42 (21.4%) controls (odds ratio [OR], 13.87; 95% CI, 5.03–38.25; *P* < .001). Thirty of 48 (62.5%) participants identified important medical information from the SP encounter in the experimental group versus 4 of 42 (9.5%) in the control group (OR, 15.83; 95% CI, 4.84–51.76; *P* < .001). Of 48 (56.3%) participants 27 made the correct transport decision to the preferred hospital in the experimental group compared with 10 of 42 (23.8%) in the control group (OR, 4.11; 95% CI, 1.65–10.23; *P* = .02). More than three-quarters (81.1%) of all participants felt they made the appropriate transport decision, with no statistically significant difference between study arms.

Participants, on average, ranked patient identifying information as the most important piece of data, followed (in descending order) by prior medical conditions, allergies, medications, emergency contact information, preferred language, treatment facility of choice, and primary care physician contact information (Table 3 and Supplement 1, Q3).

Overall, participants reported that facial recognition technology would be better than their current procedures when dealing with noncommunicative patients, and they believed there was an overall benefit to using it for patient assessment. Likert scale questions (Supplement 1, Q2) addressing current procedures—including improvements in patient ID, treatment decision capability, identification of contraindications, and transport decision capability—showed statistically significant differences between study groups (Figure 2).

## DISCUSSION

In our study, facial recognition technology demonstrated potential for quickly identifying patients and retrieving critical information for emergent care (eg, past medical history, allergies, medications, emergency contact, and primary care physician). It increased the pertinent information available when patients had altered mental status or were unconscious.

We found statistically significant differences in study participant responses about whether the app could identify SPs and their corresponding data. The app made it possible to identify the noncommunicative SP and their corresponding medical information. In the field, facial recognition could provide access to patient data that may improve patient outcomes. Additionally, paramedics believed the app would be useful in a prehospital setting due to its ease of use and the information provided. Building a universal, robust patient database would increase the likelihood of matches when using facial recognition technology.

Unexpectedly, access to patient information did not result in statistically significant differences in treatment decisions between groups. This is most likely due to the use of urosepsis as the SP’s primary condition, something commonly encountered by paramedics. The participants followed their sepsis protocols regardless of their access to patient information. Antibiotic administration is not part of the participants’ treatment protocol for this EMS department. Many other EMS agencies are now administering antibiotics in the field,^29^,^30^ where knowledge of the patient’s antibiotic allergy could have prevented an adverse reaction. However, this would also be helpful when patient care is transferred to the hospital.

In this study scenario, where all hospital choices are equidistant, the patient’s preferred hospital was defined as the facility where they received most of their medical care and/or one within their insurance network. This was also the best transport decision in a non-life and limb-threatening emergency, as per the scenario used in this study. However, only 56.3% of the experimental group chose the correct hospital despite having access to this information, although this was not the primary objective of the study. A reason for this could be the participants’ current practice or protocol, as most EMS systems lack the ability for EMS clinicians to review a patient’s past medical history or prior encounters. Access to past medical history, medication lists, and prior ECGs could enhance EMS and hospital care in certain encounters, including the knowledge of the preferred care facility, especially for patients with complex medical histories, where the clinicians are aware of the patient. It may reduce unnecessary delays in care and decision-making.

In trauma, knowing the history of bleeding disorders or any anticoagulant use may guide transportation directly to a trauma center, skipping the need for a secondary transfer to a trauma center, hence saving time and cost for care delivery. For suspected acute coronary syndrome, past medical history with prior ECGs may allow risk stratification, potentially prioritizing high-risk patients for faster transport to percutaneous coronary intervention capable facilities. A comprehensive past medical history could also alert EMS to medication allergies or other critical underlying conditions. Whether access to these data improves outcomes across various emergencies warrants further investigation. Participants rated “choice treatment facility” as the second-least important information that could be provided to them by a patient, suggesting that the value of transport-related data may need to be better communicated to EMS clinicians.

The differences in the identification of medical information between study arms suggest that facial recognition technology could reduce the likelihood of harmful interventions in these complex situations, although this warrants evaluation in additional contexts. Evaluating the technology in a mass casualty scenario could further inform its usefulness in situations where patient misidentification and medical treatment errors are common. In case of a false negative—where a field image fails to match the enrolled individual—clinicians would follow existing protocols and attempt additional images under different lighting conditions, angles, and positions.

The facial recognition app used in this study holds FirstNet Certification from the US government, which requires verification that the app is relevant to first responders’ needs, meets security and data protection standards, and maintains a documented history of system availability. The app also incorporates several technical safeguards against false matches, including similarity score thresholds, return of both field and enrolled images for manual inspection, supplementary identifying images, and emergency contact information to allow independent confirmation.

First responders are expected to handle information provided by this app consistent with standard patient chart access protocols. The unique aspect of using this technology is that this information is now available to first responders in an emergency. However, they are expected to handle this information in accordance with Health Insurance Portability and Accountability Act guidelines.

## LIMITATIONS

This double-blind, randomized control study had some limitations. The study timeline was truncated because a spike in COVID-19 cases delayed its start. We asked participants to self-report patient identification and important medical information rather than have them answer in a way that could be confirmed, to reduce the likelihood of forgetting or cheating. There is a possibility that participants may have inferred group allocation based on the app’s match output, introducing potential performance bias; this risk was mitigated, although not eliminated, by blinding and physical separation of evaluators, identical tablet access and instructions in both arms, independent SP encounters, and use of an objectively scored primary outcome.

Finally, the study relied on a simulation in which all SPs had their images intentionally uploaded into the facial recognition database. In real-world settings, an individual’s face would only be identifiable if they had enrolled in the system or if their image had been added through a participating healthcare institution (after obtaining consent). Not all patients encountered in the field would therefore be immediately identifiable; also, facial disfigurement due to burns or other traumatic injuries, along with malfunctioning of the tablet, can limit the identification, and the potential for false negatives must be acknowledged. The technology does not imply that all individuals are inherently “in the app’s cloud database,” and its utility depends on the breadth and completeness of the enrolled population.

## CONCLUSION

This pilot study demonstrated that using facial recognition technology to manage standardized patients in a simulated EMS scenario improved patient identification, access to vital patient information, and transport decisions, and was found to be beneficial by participants. Access to such critical information (eg, allergies, medications) at the point of care can directly improve patient safety. Future studies should examine the technology’s impact on scene time, triage, and destination decisions in diverse EMS systems. Additional exploration could focus on the technology’s utility across various emergency conditions (eg, trauma and stroke), and its possible effects on resource utilization.

## Supplementary Information



## Figures and Tables

**Figure 1:**
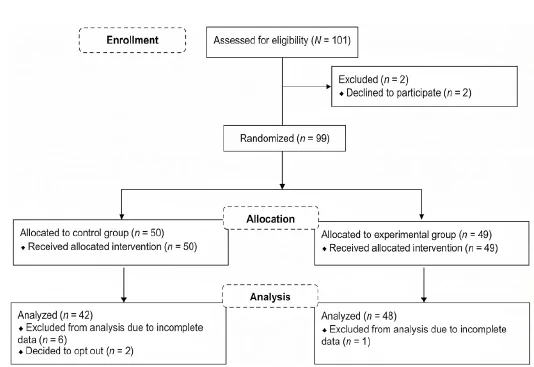
Study design flowchart illustrating participant enrollment, allocation, and exclusions in a randomized controlled simulation study comparing paramedics’ ability to identify noncommunicative patients and make related clinical decisions when using a cloud-based facial-recognition app vs standard practice.

**Figure 2:**
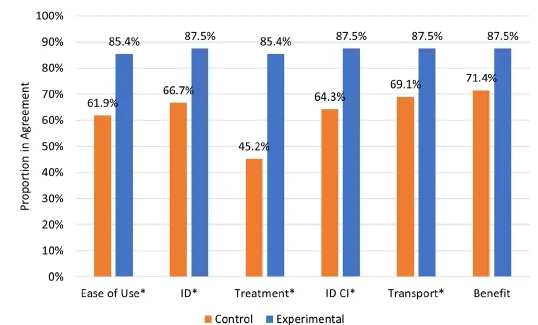
Proportion of participants agreeing with statements about the usability and clinical utility of the facial-recognition application in a study comparing paramedics’ ability to identify noncommunicative patients and make related clinical decisions when using a cloud-based facial-recognition app vs standard practice. *ID*, identification; *ID CI*, identification of contraindications,

**Table 1:** Demographic characteristics of participants in a randomized controlled simulation study comparing paramedics’ ability to identify noncommunicative patients and make related clinical decisions when using a cloud-based facial-recognition app vs standard practice.

Category	All Count (%)	Control Count (%)	Experimental Count (%)
	90 (100.0%)	42 (46.7%)	48 (53.3%)
Age	*P* = .23		
< 30 years	16 (17.8%)	9 (21.4%)	7 (14.6%)
31–40 years	38 (42.2%)	21 (50.0%)	17 (35.4%)
41–50 years	25 (27.8%)	8 (19.1%)	17 (35.4%)
≥ 51 years	11 (12.2%)	4 (9.5%)	7 (14.6%)
Sex	*P* = .18		
Male	80 (88.9%)	39 (92.9%)	41 (85.4%)
Female	9 (10.0%)	2 (4.8%)	7 (14.6%)
Prefer not to say	1 (1.1%)	1 (2.4%)	0 (0.0%)
Race	*P* = .86		
White	71 (78.9%)	33 (78.6%)	38 (79.2%)
Black	12 (13.3%)	5 (11.9%)	7 (14.6%)
Other	7 (7.8%)	4 (9.5%)	3 (6.3%)
Hispanic	***P*** = .56		
No	24 (26.7%)	10 (23.8%)	14 (29.2%)
Yes	66 (73.3%)	32 (76.2%)	34 (70.8%)
Highest level of education	***P*** = .24		
High school diploma or equivalent	15 (16.7%)	10 (23.8%)	5 (10.4%)
Associate	45 (50.0%)	20 (47.6%)	25 (52.1%)
Bachelor	23 (25.6%)	8 (19.1%)	15 (31.3%)
Graduate	7 (7.8%)	4 (9.5%)	3 (6.3%)
Years of experience	*P* = .22		
< 5 years	9 (10.0%)	3 (7.1%)	6 (12.5%)
6–15 years	45 (50.0%)	25 (59.5%)	20 (41.7%)
≥ 16 years	36 (40.0%)	14 (33.3%)	22 (45.8%)

**Table 2:** Participant responses to evaluator-assessed outcomes (1 = strongly disagree to 5 = strongly agree) in the treatment vs control group.

Variable	Mode Control (min 1, max 5)	Mode Experimental (min 1, max 5)	OR (95% CI)	*P* value
Patient identification	1	5	13.87 (5.03–38.25)*	< .001*
Patient contraindication identification	1	5	15.83 (4.84–51.76)*	< .001*
Treatment confidence	5	5	2.19 (0.72–6.65)	.16
Transport confidence	5	5	2.48 (0.83–7.44)	.09
Transport**	--	--	4.11 (1.65–10.23)*	.02*

Binary hypothesis testing of outcome variables by study arm, including odds ratio.

* Indicates statistical significance at α = .05.

** indicates results derived from questions asked by evaluators.

*OR*, odds ratio.

**Table 3:** Ranking of information importance (1 = most important, 8 = least important) and proportion of total number (%) when identifying and managing noncommunicative patients during the encounter.

Variable	Mode Control (*n* = 42)	Mode Control (%)	Mode Intervention (*n* = 48)	Mode Intervention (%)
Identifying Information	1	35.7	1	58.3
Prior Medical Conditions	1	41.5	3	25.0
Allergies	3	34.1	3	29.2
Medications	3	35.7	5	27.1
Emergency Contact Information	5	28.6	4 and 6	20.8 each
Preferred Language	8	26.2	7	25.0
Choice Treatment Facility	7	40.5	7	33.3
Primary Care Physician/Clinician Contact Information	8	52.4	8	70.8
