# Supplementary material for: Prehospital Facial Recognition Technology: Randomized Controlled Simulation Study
Source: West J Emerg Med. 2026 Oct 29;27(6):1652–9. doi: 10.5811/westjem.53140 (PMC13761886; doi:10.5811/westjem.53140)
Supplement: Supplementary file 1 [file wjem-27-1652-s001.pdf]

Evaluating the Impact of Facial Recognition for Accessing Patient Information in Triage and Treatment

Standardized Patient Scenario Evaluation

Instructions: This questionnaire is a series of statements about your experience during the scenario. There are no correct or incorrect answers. Please indicate your own feelings about each statement below by marking the numbers that best describe your attitude or beliefs. Please be truthful and describe your attitude as it really is, not what you would like it to be. This is anonymous, with the results being compiled as a group, not individually. If you have any questions or concerns, please let the research assistant know.

In this questionnaire, "facial recognition software" refers to the app you used to obtain real-time access to the personal, medical, and emergency contact information.

A non-communicative patient is defined as one who is unable to provide information such as their identity, medical conditions, medical history, medications, allergies, or emergency contact information. This may be the result of lack of consciousness, language barriers, lack of knowledge of this information, or inability to impart the information.

Q 1. Participant Study ID Number

____

Q. 2 Please rate the following items: reflect on the encounter and provide your responses below.
(Mark your response using the scale: Strongly disagree to Strongly agree)

	Strongly Disagree (1)	Disagree (2)	Neither Agree nor Disagree (3)	Agree (4)	Strongly Agree (5)
I was able to identify the patient	○	○	○	○	○
I made appropriate treatment decisions with the information available to me	○	○	○	○	○
I was able to identify the patient's contraindications	○	○	○	○	○

	Strongly Disagree (1)	Disagree (2)	Neither Agree nor Disagree (3)	Agree (4)	Strongly Agree (5)
I made the appropriate transport decision with the information provided	O	O	O	O	O
The facial recognition software was easy to use	O	O	O	O	O
The facial recognition software improved my ability to make appropriate patient care decision(s) in the scenario	O	O	O	O	O
The facial recognition software would help me better identify non-communicative patients than the protocol I currently use	O	O	O	O	O
The facial recognition software would help me better identify non-communicative patients' contraindications than the protocol I currently use	O	O	O	O	O
The facial recognition software would help me make better transport decisions for non-communicative patients than my current protocol	O	O	O	O	O
I would benefit from having facial recognition software when evaluating patients	O	O	O	O	O

Q3. Please rank how important each of the following pieces of patient information is to you from 1 (highest rank) to 8 (lowest rank).

- Identifying information (name, sex, date of birth, address, etc.)
- Allergies
- Medications
- Emergency contact information
- Preferred language
- Prior medical conditions
- Choice treatment facility
- Primary care provider contact information

Q 4. Comments, suggestions, or feedback related to the program.

Q 5. Years of experience as a paramedic:

- ○ 5 years or less
- ○ 6 to 15 years
- ○ 16 years or more

Q 6. Age:

- ○ 30 years or less
- ○ 31 years to 40 years
- ○ 41 years to 50 years
- ○ 51 years to 60 years
- ○ 61 years or more

Q 7. Highest level of education:

- ○ High School Diploma, or equivalent
- ○ Associate
- ○ Bachelor
- ○ Master
- ○ Doctorate

Q 8. Sex:

- ○ Female
- ○ Male
- ○ Prefer not to say

Q 9. Race:

- ○ White
- ○ Black or African American
- ○ American Indian or Alaska Native
- ○ Asian
- ○ Native Hawaiian or Pacific Islander
- ○ ________ Other

Q 10. Are you of Hispanic, Latino, or Spanish origin?

- ○ Yes
- ○ No
